# A comprehensive investigation into the genetic relationship between music engagement and mental health

**DOI:** 10.1038/s41398-023-02308-6

**Published:** 2023-01-19

**Authors:** Laura W. Wesseldijk, Yi Lu, Robert Karlsson, Fredrik Ullén, Miriam A. Mosing

**Affiliations:** 1 Department of Neuroscience, Karolinska Institutet, Stockholm, Sweden; 2 Department of Psychiatry, Amsterdam UMC, location University of Amsterdam, Amsterdam, Netherlands; 3 Department of Cognitive Neuropsychology, Max Planck Institute for Empirical Aesthetics, Frankfurt am Main, Germany; 4 Melbourne School of Psychological Sciences, Faculty of Medicine, Dentistry, and Health Sciences, University of Melbourne, Melbourne, Australia; 5 Department of Medical Epidemiology and Biostatistics, Karolinska Institutet, Stockholm, Sweden

**Keywords:** Genetics, Psychiatric disorders

## Abstract

While music engagement is often regarded as beneficial for mental health, some studies report higher risk for depression and anxiety among musicians. This study investigates whether shared underlying genetic influences (genetic pleiotropy) or gene-environment interaction could be at play in the music-mental health association using measured genotypes. In 5,648 Swedish twins with information on music and sport engagement, creative achievements, self-reported mental health and psychiatric diagnoses based on nationwide patient registries, we derived polygenic scores for major depression, bipolar disorder, schizophrenia, neuroticism, sensitivity to environmental stress, depressive symptoms and general musicality. In line with phenotypic associations, individuals with higher polygenic scores for major depression and bipolar disorder were more likely to play music, practice more music and reach higher levels of general artistic achievements, while a higher genetic propensity for general musicality was marginally associated with a higher risk for a depression diagnosis. Importantly, polygenic scores for major depression and bipolar remained associated with music engagement when excluding individuals who experienced psychiatric symptoms, just as a genetic propensity for general musicality predicted a depression diagnosis regardless of whether and how much individuals played music. In addition, we found no evidence for gene-environment interaction: the phenotypic association between music engagement and mental health outcomes did not differ for individuals with different genetic vulnerability for mental health problems. Altogether, our findings suggest that mental health problems observed in musically active individuals are partly explained by a pre-existing genetic risk for depression and bipolar disorder and likely reflect horizontal pleiotropy (when one gene influences multiple traits), rather than causal influences of mental health on music engagement, or vice versa (referred to as vertical pleiotropy).

## Introduction

While, intuitively, people tend to think that music engagement (singing or playing an instrument) is good for their mental health, the association between music engagement and mental health is in reality complex (see for review: Gustavson, Coleman [1]). On the one hand, multiple studies report that people who are engaged in music or theatre report better physical and mental health [2–4]. Especially with regard to well-being and social connectedness, music engagement seems to be beneficial [5–8]. Additionally, music therapy or music interventions in clinical settings can have positive effects on patient’s mental health (see for example [9]). On the other hand, some research suggests an association in the opposite direction. Two recent studies in large population-based samples reported more depression and anxiety among professional musicians [10] and depressive, burnout and psychotic-like experiences among amateur musicians [11] compared to non-players. Furthermore, higher rates of neuroticism have been found among professional musicians compared to amateurs and non-musicians [12] and neuroticism is a known risk factor for mental health problems [13]. Additionally, music engagement is a form of creative expression and higher creativity has been associated with psychotic-like experiences [14].

There are reasons to believe that the negative associations between music engagement and mental health arise because of underlying shared genetic influences. Individuals with a higher genetic risk for schizophrenia or bipolar disorder are more likely to be a member of a creative society or work in a creative domain (both include musicianship) [15]. In line with this, we have also found that the higher risk for depressive, burnout, and psychotic-like experiences in people playing music diminishes when controlling for familial liability [11]. The presence of shared underlying genetic aetiology or *genetic pleiotropy* for music engagement and mental health can be due to several scenarios. One possibility is that one gene influences multiple traits. It is well known that genetic factors are involved in both mental health [16] and musicality [17, 18], and these genetic factors for music engagement and mental health could partly overlap. Secondly, underlying shared genes can also work via a causal effect of music engagement on mental health problems or vice versa. For example, individuals experiencing mental health problems as a result of a higher genetic risk for such problems in the first place, may be more likely to seek out environments where they can engage in musical activities to alleviate their mental health problems. In this scenario, genes influencing mental health problems will also have an (indirect) influence on music engagement, because of the causal effect of genetically predisposed mental health problems on music engagement. The first scenario has previously been referred to as ‘horizontal’ pleiotropy and the second as ‘vertical’ pleiotropy [19]. Importantly, neither scenario excludes the possibility that, under some circumstances, music engagement may have a causal positive or therapeutic effect on individual’s mental health, for example, in a clinical setting. However, in line with findings from Power, Steinberg [20] on creativity and psychopathology, we would still expect musically active individuals to have a greater genetic risk for mental health problems than individuals not involved in music.

Furthermore, more complex gene-environment interplay could also be at play in the music-mental health relationship. Potential positive effects of music engagement could depend on individuals’ genotype (gene-environment interaction). For example, music engagement could be particularly beneficial for individuals with a high genetic risk for mental health problems and have less of an effect on individuals with a low genetic risk, or vice versa. This could mask associations and explain previous mixed findings. A recent study has tested gene-environment interaction in the context of effects of sport engagement on depression and found that individuals across all levels of genetic vulnerability for major depressive disorder (MDD), including individuals at highest genetic risk, experience less episodes of depression when physically active, implying no gene-environment interaction [21]. To our knowledge, no earlier research made use of measured genetic variants to investigate gene-environment interaction in the music-mental health relationship.

The overall aim of the present study was to comprehensively investigate the basis of the association between music engagement and various mental health problems utilizing a sample of genotyped twins with information on music engagement and mental health diagnoses (self-reported and registry based). For this purpose, we made use of polygenic scores, which assess the genetic risk in individuals for a trait of interest and are calculated as a weighted count of associated alleles identified in earlier genome-wide-association studies on that trait (GWASs) [22]. It is important to note that many common genetic variants with incrementally small effects influence human behavior, requiring extremely large GWAS samples to detect all. In addition, current GWASs do not incorporate rare genetic variants. Therefore, polygenic scores as yet capture only a fraction of the expected genetic variation underlying behavioral traits, and have small effect sizes [23]. We were especially interested in polygenic scores for mental health problems previously associated with musicianship and creativity, namely MDD, bipolar disorder, schizophrenia, neuroticism, sensitivity to environmental stress, and depressive symptoms. First, to investigate the presence of shared underlying genetic aetiology between music engagement and mental health, we tested whether polygenic scores for the aforementioned mental health problems predict playing music, the intensity of music practice and the level of music achievements (amateur or professional). Additionally, as control, we tested whether the polygenic scores are associated with general artistic achievements as established by Power, Steinberg [20], scientific achievements, as well as playing sports and the intensity of sport practice. We also examined whether a genetic predisposition for general musicality, including all aspects of human interaction with music, including music production skills and achievements as well as music engagement and listening [24, 25] predicted receiving a clinical diagnosis of depression, anxiety, schizophrenia, bipolar disorder, stress-related disorders, or self-reported depressive symptoms, psychotic-like experiences or neuroticism. Lastly, to investigate gene-environment interaction, we tested whether the association between music engagement and mental health outcomes differed across levels of measured genetic risk for mental health problems.

## Methods

### Participants

The Study of Twin Adults: Genes and Environment (STAGE) is a cohort of around 32,000 twin individuals born between 1959 and 1985 registered at the Swedish Twin Registry (STR). In 2012 and 2013, 11,543 twins from this cohort completed a web survey on, among other things, music engagement, music practice and achievements in various domains (e.g., music, theatre, and science) as well as on their mental health, sport engagement and practice [26, 27].

In 2019, data from the twins from the full STAGE cohort were linked to records of the National Patient Register (NPR), the nationwide covering health care system in Sweden. The NPR includes an in-patient register (IPR) and out-patient register (OPR) [28]. The IPR contains information about hospitalizations since 1964 (with sufficient national coverage since 1977), while the OPR fully covers outpatient visits since 2001.

Also in 2019 and 2020, individuals from the STAGE cohort, who provided saliva samples between 2006 and 2008, were genotyped. After quality control, genotype data were available for 8,343 individuals of the full STAGE cohort of which 5,648 completed the web survey in 2012/2013. Members of the STR consent to linkage of their genetic, phenotypic and registry-health data. The study and analyses of biomarkers were approved by the Regional Ethics Review Board in Stockholm (Dnrs 2011/570-31/5, 2018/960-31/2) and the Swedish Ethical Review Authority (Dnr 2019-05879).

### Measures

#### Music-related measures

*Music engagement*. Participants indicated whether they had ever played a music instrument (including singing) and, if so, at what age they started, whether they still played and, if not, at what age they ceased being musically active. From this data, we constructed a binary variable representing whether the participant was musically active at the time of phenotypic data collection or not. The latter category included those that had never been musically active, as well as those that had played previously but stopped.

*Amount of music practice*. Participants that ever played a music instrument (or sang) were asked about the intensity of weekly practice during four age intervals (ages 0–5, 6–11, 12–17, and 18 years until time of measurement) and based on starting and ending age of when they played music, a measure of their lifetime amount of music practice was calculated. Individuals that never played were assigned a lifetime practice of zero.

*Music achievements*. Music achievements were measured with a Swedish version of the Creative Achievement Questionnaire (CAQ) that assesses creative achievements in different domains [29, 30] on a seven-point Likert scale ranging from 1 (‘I am not engaged in music at all’), via 4 (‘I have played or sung, or my music has been played in public concerts in my home town, but I have not been paid for this’), to 7 (‘I am professionally active as a musician and have been reviewed/featured in national or international media and/or have received an award for my music activities’).

#### Control measures

*Artistic and scientific achievements*. Just as music achievements, general artistic and science achievements were measured with the Swedish version of the CAQ [31]. In a similar matter to individuals rating their music achievements on a seven-point scale, individuals were also asked to rate their dance, writing, theater, visual arts and design, invention or construction and science research lifetime achievements. As in Power et al., 2015b, we calculated individuals’ score of general artistic achievements by combining the music, dance, writing, theater and visual arts score and individuals’ scientific achievements by combining the invention and science score.

*Sport engagement*. Participants reported on whether they ever actively trained or competed in a sports domain (excluding fitness training or physical activity in general) and, if so, at what age they started, whether they still played and, if not, at what age they stopped. As for music, we constructed a binary variable representing whether the participant was active in sports at present or not.

*Amount of sport practice*. As for music practice, data on average intensity of sports practice was provided for four age intervals, which were similarly used to calculate a measure of lifetime cumulative amount of sports practice. Individuals that never played were assigned a lifetime sports practice of zero.

#### Mental health outcomes

*Registry-based mental health outcomes*. Data on clinical diagnoses for depression, anxiety disorder, schizophrenia, bipolar disorder, or a stress-related disorder according to *the International Classification of Diseases* (ICD) after any inpatient or outpatient visit were extracted from the Swedish National Patient Registers (see [11] for more information on the extracted ICD codes). For each individual, information on if they ever received a diagnosis and, if so, when, were derived.

*Self-reported mental health. Depressive symptoms* were measured with the depression scale of the Hopkins Symptom Checklist. This scale contains six items ranging from 0 (‘not at all’) to 4 (‘extremely’), measuring depressive symptoms experienced by the individual in a work-related context in the last week, with higher scores indicating more depressive symptoms. *Psychotic-like experiences* were measured with the Community Assessment of Psychic Experiences (CAPE) questionnaire. The score is based on the individual’s response to ever feeling any of the 20 positive symptom items that can be answered with four different symptom frequency levels, from 1 (‘never’) to 4 (‘almost always’). Higher scores indicate more psychotic-like experiences. *Neuroticism* was measured using the Swedish version of the 44-item Big Five Inventory (BFI) [32, 33], where participants respond to statements ranging from 1 (‘does not agree at all’) to 5 (‘exactly’), with higher scores indicating more neuroticism.

#### Genetic data processing

A total of 8,442 twins from the STAGE cohort were genotyped using the Illumina Infinium assay (chip GSAMD-24v1-0_20011747_A1). After quality control, the sample consisted of 8,343 twin individuals, out of which 5,648 individuals provided information on music engagement. A principal component analysis was performed in the full study sample (*N* = 8,343) to generate ancestry covariates [34]; the first 10 principal components (PCs) were included as covariates in all association analyses. We also calculated PCs in the reduced sample with musical phenotypes available (*N* = 5,648), and including those as covariates in the association analyses did not change the results. For more details about the genetic data processing see Wesseldijk, Abdellaoui [25].

#### Polygenic score calculation

Polygenic scores (PGSs) are a weighted sum of each individual’s trait-associated alleles at each single nucleotide polymorphism (SNP) multiplied by that SNP’s estimated effect size on a phenotype as detected by a GWAS [22]. PGSs were calculated utilizing the most recent GWAS summary statistics available for MDD [35], bipolar disorder [36], schizophrenia [37], neuroticism, sensitivity to environmental stress (SESA) and depressive symptoms [38], and self-reported beat synchronization [24]. PGSs derived from the latter GWAS were shown to be a good proxy for genetic variation underlying general musicality in our recent validation study [25]. There was no overlap between the individuals in our current (target) sample and the discovery GWAS samples, which could lead to overestimation of the genetic predisposition of a trait [39].

To create PGSs for the 8,343 individuals in the target sample, we extracted a restricted set of common, well-imputed 1,265,094 HapMap 3 SNPs from their genotype data [40, 41]. Effect sizes as provided by the summary statistics were first re-estimated using the summary-data, based on the best linear unbiased prediction (SBLUP) approach [42, 43]. This approach computes effect sizes with best linear predictor properties that account for linkage disequilibrium between SNPs. As a reference sample for the linkage disequilibrium, a random sample of 11,064 unrelated individuals was extracted from a set of 1,246,531 HapMap 3 SNPs that passed quality control in the UK Biobank sample [44]. PGSs were generated, based on these re-estimated effect sizes, for the 8,343 individuals that passed quality control, using PLINK 1.9. Scores were then imputed for MZ co-twins (*N* = 1,350) and together with the 10 PCs merged with the phenotypic data.

All variables, with the exception of the PCs were standardized.

### Statistical analyses

All analyses were performed in STATA [45].

#### Phenotypic associations between music-related measures, control measures and mental health outcomes

To examine the effect of music engagement on receiving a registry-based mental health diagnosis, we performed survival analyses, i.e., Cox proportional hazard regressions. The present sample (*N* = 5,648) is part of the larger STAGE sample (*N* = 11,543), for which some phenotypic associations have already been reported elsewhere (see [11]). In addition, here we also investigate effects of amount of music practice, and control variables (i.e., artistic and scientific achievements and sport engagement) on the mental health diagnoses. In survival analyses, the time until an event happens is used as outcome variable. We used the time (years) from the age of twelve to either the date of first receiving the mental health diagnosis or the date of censoring (i.e., date of death or end of follow-up at January 1, 2017) as the time scale (i.e., the survival time). Thus, age is accounted for in the survival model. Separate survival models were fitted for each predictor and outcome (depression, anxiety, schizophrenia, bipolar and stress-related disorders). As music-related measures we investigated music engagement (does not play music (anymore) versus plays music, 0/1), amount of lifetime music practice and level of music achievements. As control measures, we investigated general artistic achievements, scientific achievements and sport engagement (not active (anymore) versus still active, 0/1) and lifetime amount of sport practice. A hazard ratio (HR) greater than one indicates an increased risk to be diagnosed with the mental health disorder, while a value below one indicates a protective effect against the diagnosis.

We further conducted several linear regression analyses with music engagement, music practice, music achievements, general artistic achievements, scientific achievements, sport engagement or sport practice as independent variable and a self-reported continuous mental health outcome as dependent variable (i.e., depressive symptoms, psychotic-like experiences or neuroticism).

#### Associations between genetic risk for mental health problems and music-related measures

First, to validate the PGSs, we fitted the above-described survival and linear regression analyses with PGSs of MDD, bipolar disorder, schizophrenia, neuroticism, SESA and depressive symptoms as predictors and registry-based mental health diagnoses or self-reported mental health problems as the outcome. Next, we used logistic and linear regression analyses to test whether PGSs of MDD, bipolar disorder, schizophrenia, neuroticism, SESA and depressive symptoms predicted individuals’ music-related behavior. Specifically, we tested if an individual’s genetic risk for mental health problems predicts whether they play music or not (anymore), how much they practice music or their level of music achievements.

To distinguish between horizontal and vertical pleiotropy in the observed associations, we examined whether associations remained of similar size when removing participants who ever received a mental health diagnosis (*N* = 286 depression or bipolar), while adding self-reported depressive symptoms as a covariate. In case the association disappears, the manifestation of clinical symptoms seems to explain the association, implying that vertical pleiotropy (i.e., causality or genetic mediation) could be at play. If the association remains, horizontal pleiotropy is the more likely scenario, as an individual’s genetic vulnerability for mental health problems, even without suffering from symptoms, still predicts music engagement.

#### Associations between genetic risk for mental health problems and control measures

For the sake of comparison and to replicate findings from Power et al. (2015), we conducted linear and logistic regression analyses to test whether PGSs of MDD, bipolar disorder, schizophrenia, neuroticism, SESA or depressive symptoms predicted individuals’ level of general artistic and scientific achievements as well as sport engagement and amount of lifetime sport practice.

#### Associations between a genetic propensity for general musicality and mental health outcomes

We applied survival analyses to test whether the PGS for general musicality predicted a registry-based diagnosis of depression, anxiety disorder, schizophrenia, bipolar disorder or stress-related disorder. We used linear regressions to test whether the PGS for general musicality (independent variable) predicted self-reported continuous mental health outcomes (dependent variables).

Again, to further distinguish between horizontal and vertical pleiotropy, we tested the predictive value of the PGS while adjusting for lifetime music practice. If the effect remains, horizontal pleiotropy is the more likely scenario, as a genetic propensity for general musicality, even without practicing music, still predicts mental health outcomes.

#### Gene-environment interaction between genetic risk for mental health problems and music activity on mental health outcomes

Lastly, we investigated whether the effect of music engagement or music practice on mental health outcomes varied depending on an individuals’ PGS for mental health problems, by including an interaction term between music engagement and the PGS of interest. Specifically, we used survival analysis to investigate possible interactions between the PGS for MDD or bipolar and music engagement (either operationalized as a binary variable, yes/no, or as lifetime music practice) on the risk of receiving a diagnosis of depression, bipolar or anxiety disorder. Linear regression analyses were used to estimate whether there was a corresponding interaction effect on self-reported depressive symptoms.

The following applied to all of the above described models: (1) we adjusted for sex and age (either implicitly in survival models or by adding age as a covariate), and corrected for relatedness in the twin sample by using the robust standard error estimator for clustered observations [46, 47], (2) in analyses that included a PGS, we included the 10 PCs as covariates to control for ancestry structures; and (3) we repeated all PGS analyses with only one of the MZ twin members included, with results being nearly identical to in the full sample. We used an adjusted alpha of 0.05/3 = 0.016 to account for multiple testing (i.e., three overarching hypotheses/tests, namely genetic associations between mental health problems and (1) music engagement, (2) creative achievements, and (3) sport engagement). Our analyses code is available at https://osf.io/n2f36/.

## Results

The final sample for analyses concerning measured musical phenotypes consisted of *N* = 5,648 individuals (59.12% female, mean age = 40.38, SD = 7.77). The final sample used for testing associations between a genetic propensity for general musicality and mental health outcomes consisted of *N* = 9,670 (60.33% female, mean age = 40.13, SD = 7.76), as these analyses did not depend on measured musical phenotypes and included imputed MZ co-twins. See Supplementary Table S1 for descriptives of the measured phenotypes.

### Phenotypic associations between music-related measures, control measures and mental health outcomes

In line with findings from the full sample as reported in Wesseldijk, Ullén [11], none of the effects of music engagement or music practice on registry-based mental health diagnoses reached significance (see Table 1). However, music engagement and practice were associated with more self-reported depressive and psychotic-like experiences and higher levels of music achievements were associated with more self-reported psychotic-like experiences. General artistic achievements, but not scientific achievements, predicted a higher risk of receiving any registry-based diagnosis as well as experiencing more self-reported depressive and psychotic-like experiences. In contrast, the associations between sport engagement and amount of lifetime sport practice and mental health outcomes, respectively, were in the opposite direction.

**Table 1:** Hazard Ratios (HR) from survival analyses investigating the effects of music engagement (no/yes, 0/1), amount of lifetime music practice, music achievements, artistic and scientific achievements, sport engagement (no/yes, 0/1) and amount of lifetime sport practice on registry-based mental health diagnoses.

	Registry-based diagnosis of										Self-reported					
	Depression		Anxiety		Schizophrenia		Bipolar		Stress-related		Depressive symptoms		Psychotic-like experiences		Neuroticism	
N_diagnosed_	275		217		10		39		170		4,414		3,831		5,230	
	*HR*	*P-value*	*HR*	*P-value*	*HR*	*P-value*	*HR*	*P-value*	*HR*	*P-value*	*β*	*P-value*	*β*	*P-value*	*β*	*P-value*
*Music*																
Engagement	1.25	0.12	1.17	0.33	2.42	0.17	1.10	0.80	0.96	0.82	0.08*	0.04	0.12**	**<0.01**	−0.02	0.48
Practice	1.09	0.10	1.02	0.71	1.41	0.19	1.20	0.16	0.98	0.74	0.04**	**<0.01**	0.08**	**<0.001**	−0.00	0.75
Achievements	1.05	0.53	1.05	0.59	0.95	0.83	1.47*	0.05	1.08	0.40	0.02	0.14	0.06**	**<0.001**	−0.02	0.15
*Other creative achievements*																
Artistic	1.27**	**<0.001**	1.27**	**<0.001**	1.61**	**<0.01**	1.66**	**<0.001**	1.20*	0.02	0.06**	**<0.001**	0.15**	**<0.001**	0.02	0.30
Scientific	1.06	0.47	0.95	0.56	1.18	0.40	1.07	0.81	0.99	0.92	0.03	0.09	0.01	0.39	−0.03	0.10
*Sport*																
Engagement	0.92	0.55	0.73	0.07	0.64	0.58	0.40*	0.05	0.91	0.63	−0.18**	**<0.001**	−0.04	0.31	−0.25**	**<0.001**
Practice	0.87*	0.03	0.88	0.10	0.81	0.62	0.55*	0.02	0.84*	0.03	−0.10**	**<0.001**	0.01	0.67	−0.12**	**<0.001**

**Adjusted *p*-value < 0.016, *p-value < 0.05. Earlier reported results in Wesseldijk et al., 2019 for musical engagement are based on a larger sample, with linked registry-data until 2015 instead of 2017, and years were split on whether the individual did not play, stopped playing, or currently played a musical instrument which led to slightly different findings.

Number of cases receiving the diagnosis or sample sizes are reported at the top. On the right, standardized beta coefficients (β) from the linear regression analyses investigating the effects on self-reported depressive, psychotic-like experiences and neuroticism symptoms are displayed. Significant effects in bold.

### Associations between genetic risk for mental health problems and music-related measures

As expected, higher PGSs for MDD, bipolar and schizophrenia predicted a higher risk for receiving a diagnosis of the respective disorder. There were also several across-disorder predictions (see Supplementary Table S2). Further, PGSs for MDD and bipolar were associated with playing music and amount of music practice; in addition, PGS bipolar predicted music achievement, though not significantly considering an adjusted *p*-value (see Table 2, left panel). Most of these effects remained of similar size when excluding individuals with a diagnosis of depression (*N* = 275) or bipolar (*N* = 11 not comorbid with MDD) and while controlling for self-reported depressive symptoms, with the exception of the association between PGS MDD and music practice (see lower part of Table 2, left panel).

**Table 2:** Odds Ratios (OR) or standardized beta coefficients (β) for the effects of the Polygenic Scores (PGS) for mental health problems on music engagement (no/yes, 0/1), amount of lifetime music practice, music achievements, general artistic and scientific achievements, sport engagement (no/yes, 0/1) and amount of lifetime sport practice.

	Music						Achievements				Sport			
	Engagement (no/yes)		Practice		Achievements		Artistic		Scientific		Engagement (no/yes)		Practice	
N	*4,431*	*1,220*	*5,636*		*3,838*		*3,836*		*3,837*		*3,885*	*1,559*	*5,440*	
Polygenic score	*OR*	*P-value*	*β*	*P-*	*β*	*P-value*	*OR*	*P-value*	*β*	*P-value*	*OR*	*P-value*	*β*	*P-value*
MDD	1.08*	0.04	0.04**	**<0.01**	0.02	0.40	0.12*	0.04	−0.08*	0.02	0.94*	0.05	−0.04**	**<0.01**
Bipolar	1.12**	**<0.01**	0.04*	0.02	0.05*	0.05	0.15**	**0.01**	0.07*	0.03	1.01	0.67	−0.02	0.29
Schizophrenia	1.03	0.41	0.02	0.18	0.01	0.61	0.12*	0.04	0.04	0.16	1.02	0.53	0.00	0.92
Neuroticism	1.00	0.94	0.01	0.54	−0.04	0.12	−0.03	0.55	−0.14**	**<0.001**	0.90**	**<0.01**	−0.03*	0.02
SESA	1.03	0.96	0.02	0.24	0.01	0.85	0.05	0.36	−0.05	0.11	0.92**	**<0.01**	−0.05**	**<0.001**
Depressive symptoms	0.98	0.61	−0.00	0.87	−0.05	0.07	−0.05	0.37	−0.09**	**<0.01**	0.95	0.11	0.00	0.96
*Sensitivity analyses*														
*N*	*4,217*	*1,148*	*5,350*		*3,640*									
MDD	1.05	0.23	0.04*	0.04	0.01	0.66								
Bipolar	1.11**	**0.01**	0.04*	0.02	0.04	0.09								

**Adjusted *p*-value < 0.016, *p-value < 0.05. *MDD* major depressive disorder, *SESA* sensitivity to environmental stress.

Sample sizes are reported at the top. The lower part of the table displays the results of the sensitivity analyses for PGS MDD and Bipolar in a sample excluding individuals who received a depression or bipolar diagnosis (*N* = 286) while including self-reported depressive symptoms as a covariate. Significant effects in bold.

### Associations between genetic risk for mental health problems and control measures

Table 2 (right panel) shows the associations between PGSs for various mental health problems and artistic and scientific achievements, as well as sport engagement and the amount of lifetime sport practice. PGSs for MDD, bipolar and schizophrenia were associated with higher levels of general artistic achievements. Post-hoc analyses revealed that achievements in the domain of writing drove the effects of the PGSs for schizophrenia and MDD on general artistic achievements. Furthermore, PGSs for MDD, neuroticism and depressive symptoms were associated with lower levels of scientific achievements, while a PGS for bipolar disorder predicted higher levels of scientific achievements. Lastly, higher PGSs for MDD, neuroticism and SESA were all associated with a lower chance of playing sports and less sport practice.

### Associations between a genetic propensity for general musicality and mental health outcomes

As shown in Table 3, a genetic propensity for musicality predicted a higher risk of receiving a depression diagnosis, but no other mental health diagnoses or self-reported mental health problems, regardless of whether and how much individuals played music. However, this effect did not survive a correction for multiple testing.

**Table 3:** Hazard Ratios (HR) from survival analyses investigating the effect of the Polygenic Score (PGS) for general musicality on registry-based mental health diagnoses and standardized beta coefficients (β) and p-values from linear regression analyses investigating the effect of PGS music on self-reported depressive and psychotic-like experiences.

	Registry-based diagnosis of											Self-reported						
	Depression		Anxiety		Schizophrenia		Bipolar		Stress-related			Depressive symptoms			Psychotic-like experiences		Neuroticism	
N_diagnosed_	491		396		21		73		330			4,414			3,831		5,230	
Polygenic score	*HR*	*P-value*	*HR*	*P-value*	*HR*	*P-value*	*HR*	*P-value*	*HR*	*P-value*		*β*	*P-value*		*β*	*P-value*	*β*	*P-value*
Music	1.10*	0.04	1.05	0.34	0.86	0.51	1.06	0.64	0.95	0.41		−0.01	0.71		0.00	0.83	−0.01	0.38
*Sensitivity analyses*																		
Music	1.15*	0.03	1.05	0.48	0.96	0.88	1.18	0.35	1.07	0.44		−0.01	0.53		0.00	0.74	−0.01	0.41

**Adjusted *p*-value < 0.016, *p-value < 0.05.

Number of cases receiving the diagnoses or sample sizes are reported at the top.

The lower part of the table displays the results of the sensitivity analyses including amount of lifetime music practice as a covariate. Significant effects in bold.

### Gene-environment interaction between genetic risk for mental health problems and music activity on mental health outcomes

The effects of music engagement and lifetime amount of music practice on a registry-based depression, anxiety or bipolar diagnosis or on self-reported depressive symptoms as reported in Table 1, did not differ depending on an individuals’ PGS for MDD or bipolar disorder, as all interaction terms were non-significant (all *p* > .06). In other words, we found no evidence that the associations between music engagement and mental health problems differed across different levels of genetic vulnerability for mental health problems.

## Discussion

To shed light on the music-mental health association, the current study is the first to use polygenic scores to investigate the presence of shared underlying genetic influences and gene-environmental interplay for music engagement and mental health. Individuals engaged in music did not receive more registry-based mental health diagnoses, but did report more mental health problems, than individuals not engaged in music. In line with these phenotypic associations, we found that higher genetic risks for MDD and bipolar disorder were associated with playing music and more hours of music practice. Nearly all genetic associations remained of similar effect size when excluding individuals diagnosed with MDD or bipolar and controlling for self-perceived mental health problems, indicating that the effect of genetic risk for mental health problems on music engagement is unlikely driven by a manifestation of psychiatric symptoms. Additionally, a genetic propensity for general musicality appears to increase the likelihood of receiving a depression diagnosis. This effect was present regardless of whether and how much individuals played music, meaning that playing music cannot explain the effect. Lastly, we found no evidence for gene-environment interaction, associations between music engagement and mental health did not depend on individuals’ genetic risk for mental health problems.

Our findings show the presence of shared underlying genetic influences (genetic pleiotropy) for music engagement and mental health, explaining at least part of the phenotypic associations observed. As already touched upon in the introduction, two possible mechanisms, ‘horizontal’ or ‘vertical’ pleiotropy, could be at play. In the case of horizontal pleiotropy, the same genes influence both music engagement and mental health, either directly or via an intermediate variable [19]. Vertical pleiotropy involves the presence of a causal pathway between mental health and music engagement or vice versa, resulting in the genetic variants underlying one of the two traits to consequently also be associated with the other. Our findings show that a causal pathway (vertical pleiotropy) is unlikely to explain the shared genetic influences, as the associations were independent of experiencing mental health problems or music practice. Overall, this suggests that the genetic overlap likely involves horizontal pleiotropy. Importantly, we cannot exclude that mental health problems independent of genetic risk causally affect music engagement or vice versa. However, in past research using twin data, we found no support for a causal effect of music engagement on mental health [11].

Our findings on the shared underlying genetic influences for MDD, bipolar and music engagement resonates with findings from Power et al. (2015b) on schizophrenia, bipolar and creativity. Both studies found phenotypic associations to be partly explained by underlying shared genetic influences. Here, we replicated Power’s finding that genetic risks for schizophrenia and bipolar predict general artistic achievement. In addition, we found genetic risk for MDD (a predictor not included in Power et al., 2015b) to predict music engagement and general artistic achievement, but not music achievement, a domain included in the general artistic achievements measure (here and in Power et al 2015b). The association between genetic risk for MDD and general artistic achievements is mainly driven by achievements in the writing domain. This is not surprising as historically writers are known to be particularly prone to psychiatric problems, even among artistic groups [48]. Overall, genetic overlap between MDD and general artistic achievements seems to be (partly) distinct from the genetic overlap between MDD and music engagement.

As control, we also investigated genetic overlap between mental health, scientific achievements and sport engagement, which showed associations in the opposite direction than for music engagement. Genetic variants associated with MDD, depressive symptoms and neuroticism were less common among individuals active and successful in the scientific domains, while the risk for bipolar was somewhat elevated. Neither of these genetic associations were reflected in the phenotypic associations. Further, individuals who practiced more sports had a lower genetic vulnerability for MDD, neuroticism or SESA.

We found no evidence that associations between music engagement and mental health problems differed across levels of genetic vulnerability for mental health problems. The lack of gene-environment interaction implies that music engagement does not differently affect individuals at a high genetic risk for mental health problems and individuals at a low risk.

This study has some limitations. As already touched upon in the introduction, despite the mental health PGSs all significantly predicting corresponding clinical mental health diagnoses, overall PGSs explain little genetic variance (R^2’^s smaller than 2%) as they are based on GWASs that capture only a fraction of the genetic variation underlying behavioral traits. This is in line with other PGS studies, and with sample sizes of GWASs increasing, prediction accuracy of PGSs will also increase [23]. Further, we would like to point out that some of our findings did not survive a more conservative p-value of .016, highlighting the importance of future studies replicating our findings. Also, it is important to keep in mind that PGSs are not purely ‘innate’, as recent within-family GWAS and PGS methods showed that polygenic scores reflect a mix of both true causal genetic signal on the trait under study in the GWAS, but also ‘indirect’ genetic effects through passive *r*GE, i.e., effects of the association between the genotype a child inherits from its parents and the environment in which it is raised, and confounding effects, such as population stratification and assortative mating [49]. Lastly, we cannot be certain that we have sufficiently adjusted for mental health problems in our sensitivity analyses, as some participants may have gone undiagnosed or only experienced depressive problems in the past, which were not assessed in the questionnaire. Therefore, we cannot fully exclude the possibility that the actual experience of mental health problems as a result of higher genetic risk may lead to increased music engagement (i.e., vertical pleiotropy).

Altogether, this study shows that individuals engaged in music, have a pre-existing higher genetic risk for depression and bipolar disorder and that individuals with a propensity for general musicality seem to be more likely to experience depression. Such shared underlying genetic influences between music engagement and mental health most likely reflect horizontal pleiotropy rather than a causal relationship between mental health and music engagement or vice versa. Future research is needed to further disentangle the music–mental health relationship, taking into account that musicians may have a higher genetic risk for depression and bipolar disorder in the first place and that underlying shared genetic factors may confound findings.

## Supplementary information

Supplemental Material

## Data Availability

The datasets generated during the current study cannot be made publicly as registry data were used. Individuals are able to apply online at the Swedish Twin Registry to access the twin data. Our analyses code is available at https://osf.io/n2f36/.
