# Supplementary material for: A comprehensive investigation into the genetic relationship between music engagement and mental health
Source: Transl Psychiatry. 2023 Jan 19;13:15. doi: 10.1038/s41398-023-02308-6 (PMC9852421; doi:10.1038/s41398-023-02308-6)
Supplement: Supplementary file 1 — Supplemental Material [file 41398_2023_2308_MOESM1_ESM.docx]

**Supplementary Tables**

**S1.** Descriptives for the measured phenotypes.

|  | N | Range | M (SD) |
| --- | --- | --- | --- |
| Music engagement  *Active (%)* | 5,648  1,220 (21.6%) | 0-1 |  |
| Music practice | 5,639 | 0 – 27,040* | 2,411 (3,496) |
| Music achievements   1. *No playing* 2. *Played privately* 3. *Played in music lessons* 4. *Played publicly* 5. *Played and got paid for it* 6. *Played professionally* 7. *International professional player* | 2,253 (58.7%)  310 (8.1%)  400 (10.4%)  636 (16.6%)  188 (4.9%)  29 (.8%)  22 (.6%) | 1-7 | 2.06 (1.43) |
| Artistic achievements | 3,836 | 5-35 | 7.99 (3.33) |
| Scientific achievements | 3,837 | 2-14 | 3.33 (1.93) |
| Sport engagement  *Active (%)* | 5,444  1,559 (28.65%) | 0-1 |  |
| Sport practice | 5,440 | 0 – 20,696* | 3,311 (3,856) |
| Self-reported mental health  *Depressive symptoms*  *Psychotic-like experiences*  *Neuroticism* | 4,414  3,831  5,230 | 6-30  1-3.6  1-5 | 11.41 (4.79)  1.25 (.19)  2.40 (.66) |

* This is a rough approximation of lifetime hours practiced and should be considered an

indicator of relative practice compared to other participants rather than an absolute value.

**S2.** Hazard Ratios (HR) and p-values from survival analyses investigating effects of the polygenic scores for mental health problems on registry-based mental health diagnoses on the left. Number of cases receiving the diagnoses and controls are reported at the top. On the right, standardized beta coefficients (β) and p-values from the linear regression analyses investigating the effects on self-reported depressive, psychotic-like experiences and neuroticism symptoms.

|  | Registry-based diagnosis of | | | | | | | | | |  | Self-reported | | | | |  |
| --- | --- | --- | --- | --- | --- | --- | --- | --- | --- | --- | --- | --- | --- | --- | --- | --- | --- |
|  | Depression | | Anxiety | | Schizophrenia | | Bipolar | | Stress-related | |  | Depressive symptoms | | Psychotic-like experiences | | Neuroticism | |
|  | N,  cases | N,  controls | N, cases | N, controls | N,  cases | N,  controls | N,  cases | N, controls | N,  cases | N,  controls | .... |  |  |  |  |  |  |
| N | 491 | 9,179 | 396 | 9,274 | 21 | 9,649 | 73 | 9,597 | 330 | 9,320 |  |  |  |  |  |  |  |
| Polygenic score | *HR* | *P-value* | *HR* | *P-*  *value* | *HR* | *P-*  *value* | *HR* | *P-*  *value* | *HR* | *P-*  *value* |  | *β* | *P-value* | *β* | *P-value* | *β* | *P-value* |
| *MDD* | 1.43 | <.001 | 1.51 | <.001 | 1.50 | .08 | 1.77 | <.001 | 1.42 | <.001 |  | .08 | <.001 | .07 | <.001 | .11 | <.001 |
| *Bipolar* | 1.19 | <.001 | 1.15 | .01 | 1.34 | .18 | 1.82 | <.001 | 1.17 | <.01 |  | .02 | .29 | .02 | .26 | .03 | .02 |
| *Schizophrenia* | 1.22 | <.001 | 1.28 | <.001 | 1.96 | .01 | 1.59 | <.001 | 1.09 | .13 |  | .05 | <.01 | .02 | .33 | .06 | <.001 |
| *Neuroticism* | 1.36 | <.001 | 1.44 | <.001 | .97 | .87 | 1.44 | <.01 | 1.29 | <.001 |  | .11 | <.001 | .08 | <.001 | .17 | <.001 |
| *SESA* | 1.29 | <.001 | 1.35 | <.001 | 1.10 | .63 | 1.28 | .05 | 1.25 | <.001 |  | .11 | <.001 | .08 | <.001 | .14 | <.001 |
| *Depressive symptoms* | 1.20 | <.001 | 1.29 | <.001 | 1.16 | .52 | 1.42 | .001 | 1.22 | <.001 |  | .09 | <.001 | .09 | <.001 | .09 | <.001 |

MDD is major depressive disorder. SESA is sensitivity to environmental stress.
